# Supplementary figures and images for: Menopause Is Associated with an Altered Gut Microbiome and Estrobolome, with Implications for Adverse Cardiometabolic Risk in the Hispanic Community Health Study/Study of Latinos
Source: mSystems. 2022 Apr 13;7(3):e00273-22. doi: 10.1128/msystems.00273-22 (PMC9239235; doi:10.1128/msystems.00273-22)

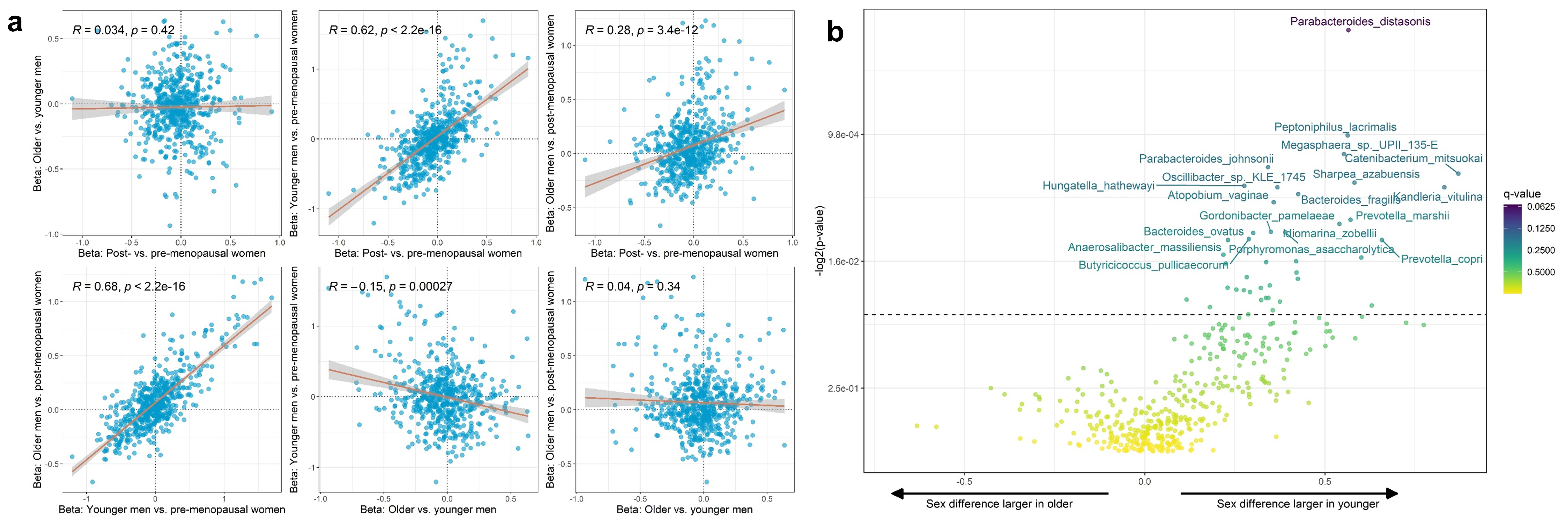

Supplement: FIG S2 [file msystems.00273-22-sf002.tif]

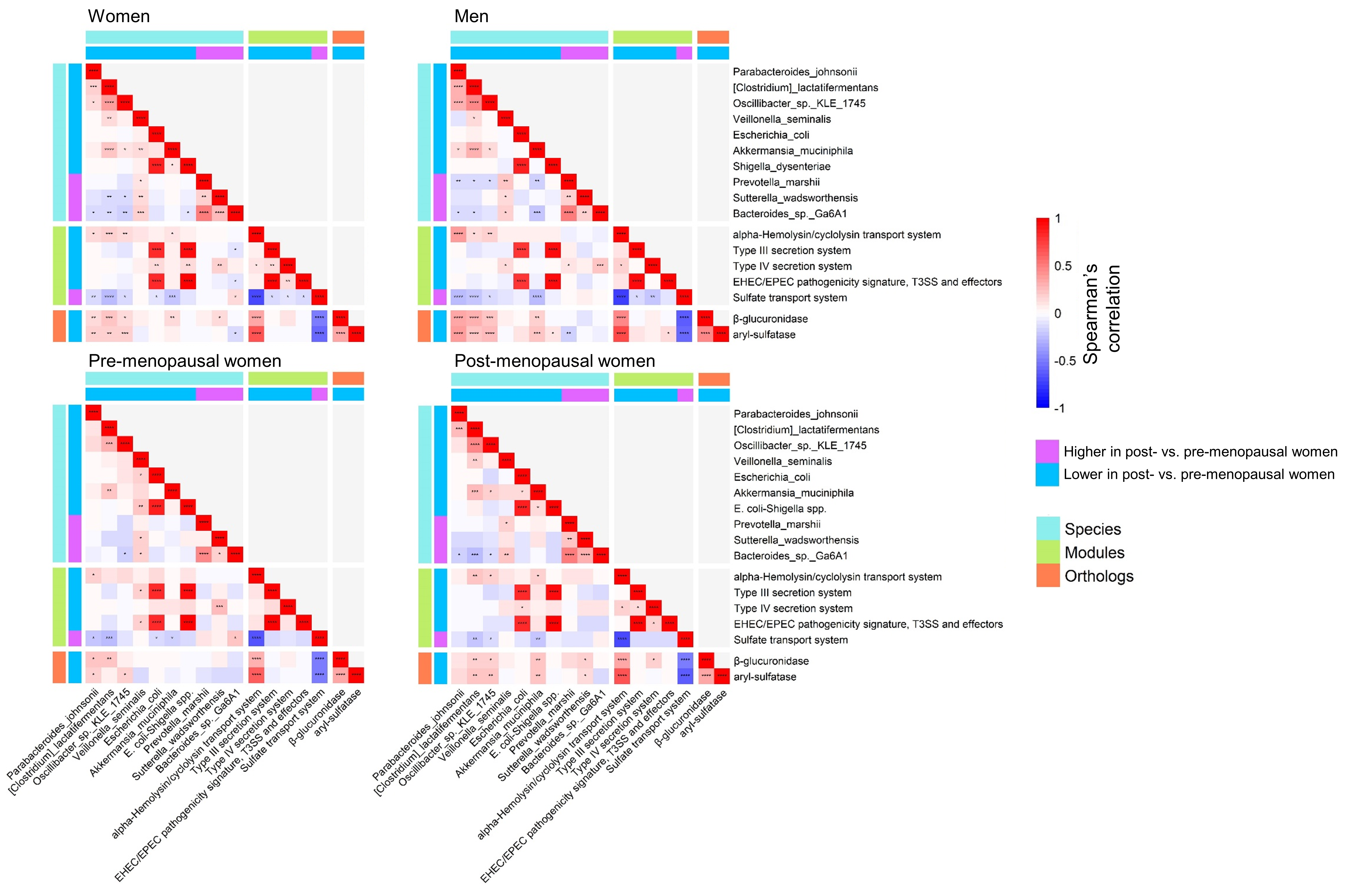

Supplement: FIG S3 [file msystems.00273-22-sf003.tif]

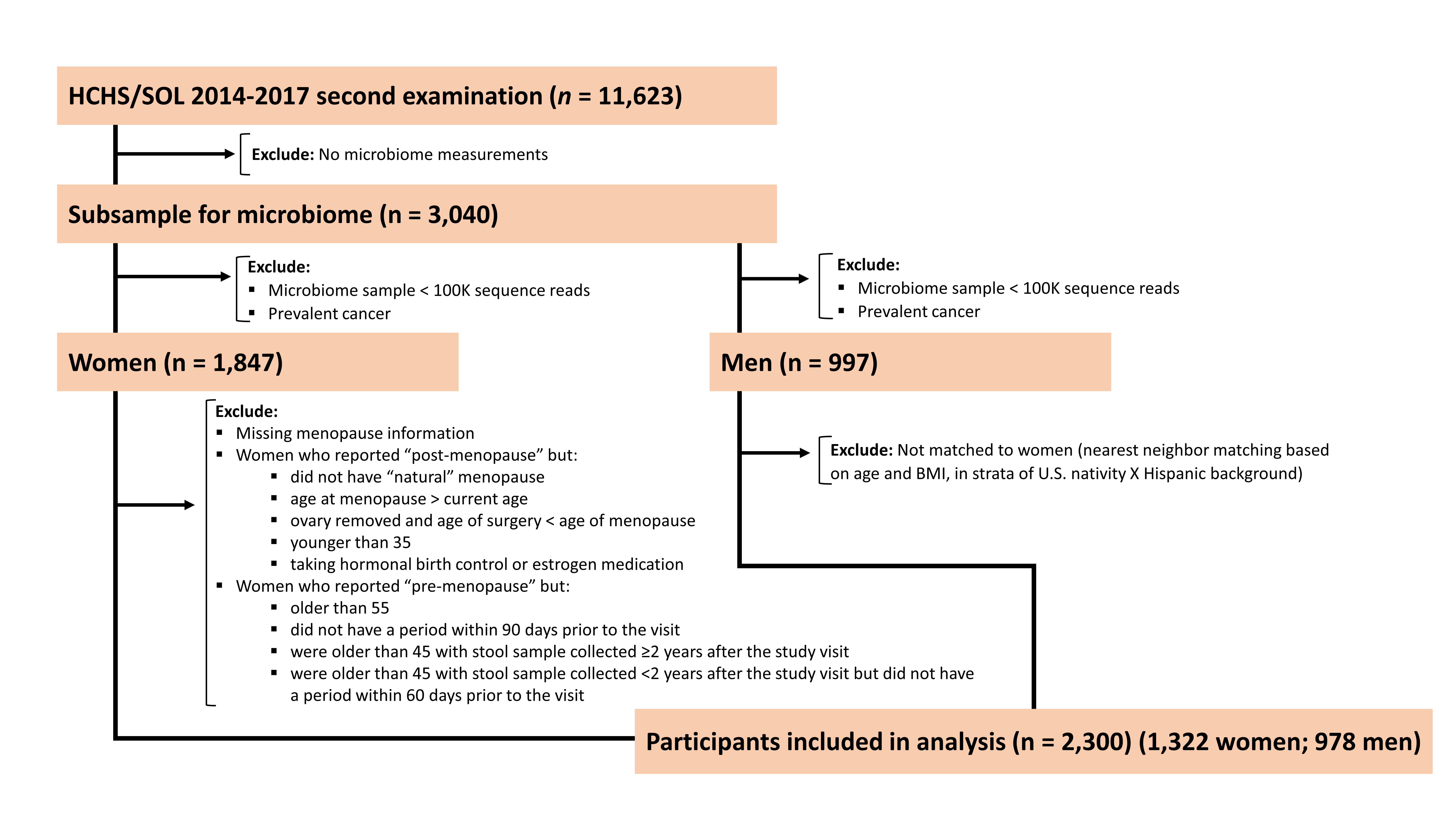

Supplement: FIG S1 [file msystems.00273-22-sf001.tif]
